# Human Urinary Volatilome Analysis in Renal Cancer by Electronic Nose

**DOI:** 10.3390/bios13040427

**Published:** 2023-03-28

**Authors:** Manuela Costantini, Alessio Filianoti, Umberto Anceschi, Alfredo Maria Bove, Aldo Brassetti, Mariaconsiglia Ferriero, Riccardo Mastroianni, Leonardo Misuraca, Gabriele Tuderti, Gennaro Ciliberto, Giuseppe Simone, Giulia Torregiani

**Affiliations:** 1 Department of Urology, IRCCS—Regina Elena National Cancer Institute, 00144 Rome, Italy; manuela.costantini@ifo.it (M.C.);; 2 Department of Urology, San Filippo Neri Hospital, 00135 Rome, Italy; 3 Scientific Direction, IRCCS—Regina Elena National Cancer Institute, 00144 Rome, Italy; 4 Department of Anesthesiology and Intensive Care Unit, IRCCS—Regina Elena National Cancer Institute, 00144 Rome, Italy

**Keywords:** electronic nose, renal cancer, tumour biomarkers, volatilome, cancer screening

## Abstract

Currently, in clinical practice there are still no useful markers available that are able to diagnose renal cancer in the early stages in the context of population screening. This translates into very high costs for healthcare systems around the world. Analysing urine using an electronic nose (EN) provides volatile organic compounds that can be easily used in the diagnosis of urological diseases. Although no convincing results have been published, some previous studies suggest that dogs trained to sniff urine can recognize different types of tumours (bladder, lung, breast cancer) with different success rates. We therefore hypothesized that urinary volatilome profiling may be able to distinguish patients with renal cancer from healthy controls. A total of 252 individuals, 110 renal patients and 142 healthy controls, were enrolled in this pilot monocentric study. For each participant, we collected, stabilized (at 37 °C) and analysed urine samples using a commercially available electronic nose (Cyranose 320^®^). Principal component (PCA) analyses, discriminant analysis (CDA) and ROC curves were performed to provide a complete statistical analysis of the sensor responses. The best discriminating principal component groups were identified with univariable ANOVA analysis. The study correctly identified 79/110 patients and 127/142 healthy controls, respectively (specificity 89.4%, sensitivity 71.8%, positive predictive value 84.04%, negative predictive value 80.37%). In order to test the study efficacy, the Cross Validated Accuracy was calculated (CVA 81.7%, *p* < 0.001). At ROC analysis, the area under the curve was 0.85. The results suggest that urine volatilome profiling by e-Nose seems a promising, accurate and non-invasive diagnostic tool in discriminating patients from controls. The low costs and ease of execution make this test useful in clinical practice.

## 1. Introduction

Renal cell carcinoma (RCC) accounts for approximately 3–4% of all new solid malignancies, with a median age at diagnosis in the sixth decade. In the United States, kidney cancer is the sixth and ninth most common cancer for men and women, respectively.

Although mortality is decreasing, worldwide the incidence of RCC has been increasing from 2009 by around a 1% rate each year, due to the widespread use of imaging tests (Ultrasounds, CT or RMI scan) performed in asymptomatic patients for other clinical reasons (stones, hypertension, backache, diabetes, etc.). An estimated of 13,000 deaths from this disease occurred in the United States in 2021 [1]. For patients with RCC localized only in the kidney, the 5-year survival rate is high (approximately 93%), while in subjects with cancer that has spread to the surrounding organs and/or regional lymph nodes the rate decreases dramatically to 60–70%. Lastly, in the case of metastatic renal disease, the 5-year survival rate becomes less than 10–14%. Despite recent advances in medical treatment, especially with targeted therapy and immunotherapy, the RCC diagnosis still has a high economic impact on national health systems worldwide [2].

Within this perspective arises the need to develop new approaches for the early diagnosis of kidney cancer using, above all, non-invasive biomarkers. Based on the recent literature, urinary Volatile Organic Compounds (VOCs) analysis has emerged as a promising field of application with the potential to develop new biomarkers for early cancer diagnosis.

In humans, VOCs are easily found in biological samples of urine, blood, breath, faeces, skin and tissue, but among of all these possible biological samples urine has the advantage of being economic to collect and simple to handle. We know that several metabolic and inflammatory pathways in the body are capable of releasing thousands of volatile organic compounds in the urine. Numerous studies have shown more than 100 urinary VOCs in the urine, mainly ketones from protein degradation. Surprisingly, some changes in the composition of urinary VOCs are associated with the presence of pathological clinical conditions such as tumours, foreign bodies and infections. For this specific reason, we can consider urinary VOCs as alternative biomarkers. Currently, however, there is still no strong evidence of a specific molecular model for renal cancer [3].

In the literature, several studies have reported that dogs trained to smell urine (called sniffer-trained dogs) are able, with various success rates, to identify different types of human neoplasms (melanoma, prostate, breast, lung and bladder cancer), but significant results have not yet been stated [4,5]. In particular, it has been reported that post specific olfactory training, dogs obtain the capacity to identify some urological tumours by smelling urine, with encouraging outcomes. Unfortunately, the difficulty of this approach lies in the poor reliability and large-scale reproducibility of these results and in the problematic employment of the dogs’ olfaction in health care settings.

To overcome these critical aspects, specific advanced technologies have been tested—especially a gas sensors array, also called Electronic Nose (EN or e-Nose). This device, being able to reproduce a dog’s sense of smell for the identification of volatile compounds in human biological fluids, could represent a promising diagnostic tool for several medical conditions [6]. The e-Nose can be described as a complex technological device consisting of several thin-film nanocomposite sensors that produce various chemical interactions, in *primis* reversible electron transfer reactions, for the correct measurement of gases, acids, bases and many other compounds. However, its operation is relatively simple: when the tool is exposed to a sample, it provides a unique smell-print which, through a specific pattern recognition system, can be analysed in order to identify its origin and nature [7].

With this prospect, the e-Nose proves to be a particularly suitable tool for the quantitative and qualitative analysis of complex gaseous molecular blends [8].

For a long time, indeed, this type of device has been applied in areas where the intrinsic analytical capacity of the instrument is indispensable: in chemical industries, agricultural and food quality controls, air or environmental pollution, safety, odour monitoring and in some military applications, for example [9,10,11,12,13,14].

Based on this, different attempts have been carried out to expand this analytical technique to various medical applications; in particular the electronic nose has proved useful in the analysis of breathed air for early cancer diagnosis. [15].

However, in clinical practice, sampling urine appears less problematic because it is easier to obtain and store, while exhaled air usually requires an immediate analysis or specific storage (in Tedlar/Nalphan bags or sorption tubes) and deep technical cooperation on the part of the patient.

To corroborate this thesis, we know that several studies in the metabolic field have analysed urine through gas/liquid chromatography–mass spectrometry techniques. Preliminary data suggest that it is possible to detect some urological malignancies using urine headspace [16]. We can therefore consider the electronic nose as a useful device for VOCs sampling, capable of identifying the mixture of VOCs and converting it into a specific urinary profile (urine smell-fingerprints). This tool provides an inexpensive, immediate, easy and on-site distinction of urine smell-prints by pattern recognition models, but it currently cannot identify individual molecular elements.

This article tests the hypothesis that renal cancer patients can be effectively distinguished from healthy controls using urine headspace analysis of VOCs by e-Nose.

## 2. Materials and Methods

### 2.1. Study Population

A total of 252 subjects participated in this pilot, monocentric, prospective study. The study cohort consisted of 2 groups: 110 renal patients (RCa group) and 142 healthy controls (HC group) enrolled at the Urology Department of Regina Elena National Cancer Institute of Rome between January 2020 and December 2021.

The presence of radiological (CT or MRI scan) evidence of renal neoplasm defined membership in the RCa group. Patients were radiologically staged using currently accepted TNM criteria, and underwent surgery. All clinically established conditions potentially able to affect the urinary VOCs spectrum were considered reasons for exclusion from the study, especially renal dysfunctions, haematuria, dysuria, urinary tract infections and lithiasis.

The control group was formed of 142 ambulatory patients undergoing a routine visit, with a negative history of urological disorders or symptoms and without evidence of any known neoplastic disease. The Internal Ethics Committee approved the study, and all subjects gave their written informed consent to participate.

### 2.2. Electronic Nose

A commercially available electronic nose (Cyranose 320^®^, Smith Detections, Pasadena, CA, USA) was used in this study. It consists of a nanocomposite array of 32 organic polymer sensors enclosed in a handheld device with a simple use: as soon as the sensors are exposed to a mixture of VOCs, the polymers expand, activating a variation in their electrical resistance [17]. This tool is equipped with an on-board database where it records raw data as changes in the resistance state of each of the 32 sensors, thus providing a distribution (urine smell-prints) that characterizes the VOC mixture and that can be easily applied in pattern-recognition algorithms. The device has a measurement chamber composed of a 40 mL polystyrene vial containing the urine sample and a Teflon soft cover top in which two holes have been drilled. The two holes are used to create two channels; the first channel is for airflow, and the second one was for the insertion of a modified 16G intravenous cannula connected to the system to provide sample air for the e-Nose. A single cannula was used during the whole study; it was sterilized after each measurement.

For each subject, a urine sample of 20 mL was collected in sterile conditions (pre-surgery in the experimental group or at the end of the ambulatory visit in the control group) and maintained at 37 °C in a water thermostatic bath. In order to prevent the decay of metabolites, including VOCs, the stabilized urine samples were immediately analysed with e-Nose. At this point, the following steps were taken: 40 s to empty sensor chamber, 30 s for sample analysis, and then the outflow acquisition. The time required for each measurement was approximately 7 min, followed by a 10 min recovery period to avoid system carryover.

The sampling was then repeated in order to obtain duplicate results.

### 2.3. Data Analysis

Raw data were initially collected from the e-Nose on-board database and subsequently analysed with SPSS software (version 20.0, SPSS Inc., Chicago, IL, USA), performing analysis strategies that deliberately limit false discoveries [18].

Data were thus reduced to a set of principal components (PCA) in order to obtain the largest amount of variance in the original 32 sensors (two-dimensional principal component analysis (2D-PCA)). Successively, univariate ANOVA analysis was performed to recognize the principal components that were best differentiated among the groups.

Finally, these principal components were used to provide a linear canonical discriminant analysis (CDA) to put cases into a categorical partition. The “leave-one-out method” was applied to estimate the Cross Validated Accuracy percentage (CVA, %), which represents an estimate of the accuracy of a predictive model in the practice.

Indeed, the CVA analysis computes a percentage that evaluates how precisely a predictive model will perform in clinical practice. The probability of a positive diagnosis was counted for each case on basis of the CDA canonical discriminant function. These probabilities were successively applied to provide a receiver operator curve (ROC curve) with a 95% confidence limit. For resistance calculation, the formula (∆R/R0) was used.

## 3. Results

The general clinical characteristics of the study population are shown in Table 1.

The two study groups (RCa and Healthy Control) were homogeneous in terms of baseline clinical data, in particular for age and comorbidities. The pathological staging of the subject belonging to the RCa group was described in Table 2. The distribution of these tumours in relation to extension (TMN) and aggressiveness (ISUP Grade) complies with other renal casistics in the literature. Moreover, we can assess that the diagnostic ability of the EN approach is not influenced by the tumour size, given that the casistics are quite varied.

**Figure 1:**
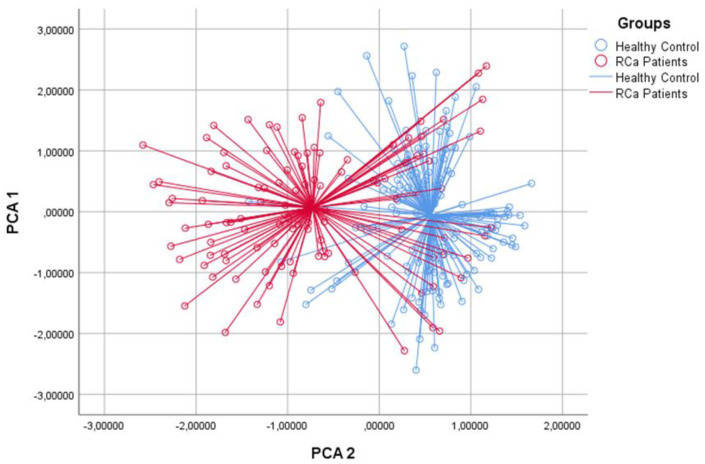
Two-dimensional Principal Component Analysis plot. The PCA plot suggests that patients with renal tumour (in red) could be well distinguished from healthy controls (in blue).

**Figure 2:**
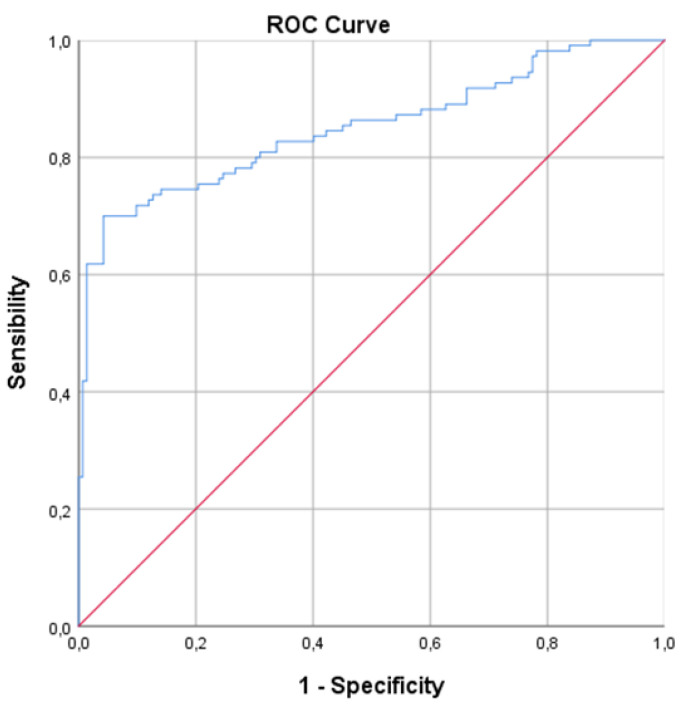
ROC Curve Analysis. In ROC analysis, the discrimination accuracy (AUC—area under the curve) between renal patients and healthy controls was 0.85. Repeated analyses with a second measure obtained from the same urine samples set provided comparable findings (CVA 81.3%, AUC 0.9, sensitivity 71.8% and specificity 88.7%).

The univariable ANOVA analysis identified the best discriminating principal component groups. Based on two-dimensional principal component analysis (2D-PCA), 79/110 and 127/142 cases were correctly identified in the RCa and healthy control groups, respectively (Figure 1). In the CDA, the CVA was 81.7% (*p* < 0.001); the sensitivity was 71.8%, specificity 89.4%, the positive predictive value (PPV) was 84.04% and the negative predictive value (NPV) 80.37% (Table 3); values that can be considered more than acceptable for the intended purpose. At the ROC analysis, the discrimination accuracy of the model (area under the curve (AUC)) was 0.85 (Figure 2).

We repeated the analyses in an independent second urine samples set, obtaining comparable findings (CVA 81.3%, AUC 0.9, sensitivity 71.8%, and specificity 88.7%).

**Figure 3:**
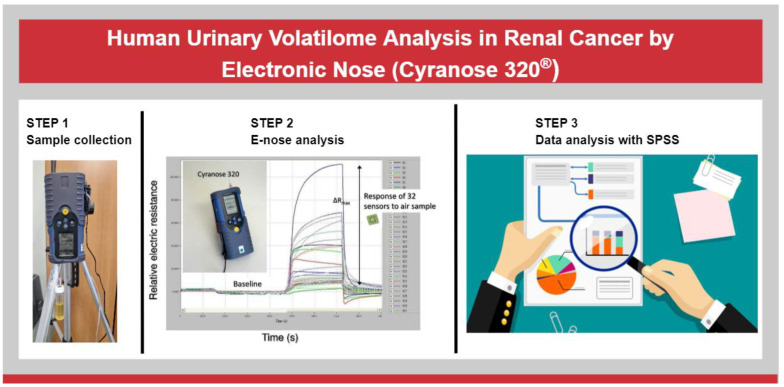
Graphical Abstract of the study. We reported a brief outline of the study, which mainly consists of three phases: collection of urine samples, processing with e-Nose and data analysis.

**Figure 4:**
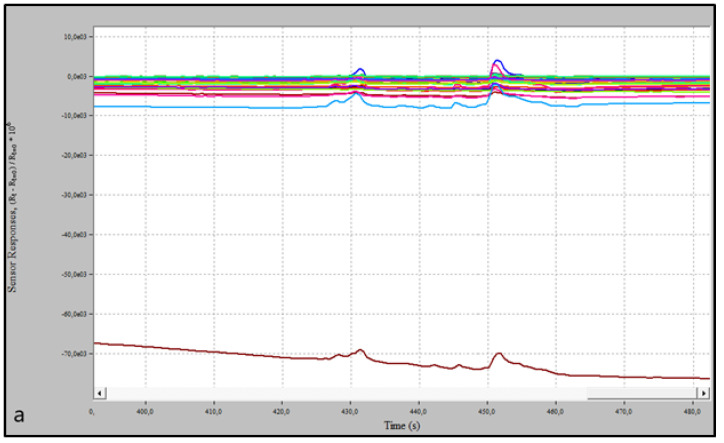

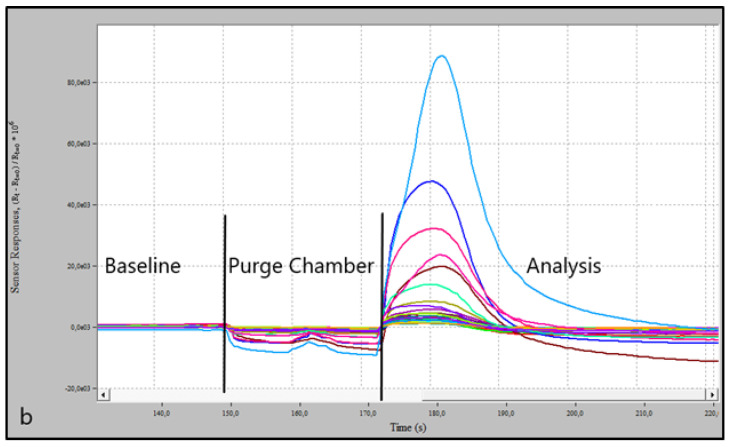
Graphical representation of the sensors responses curves during the analysis. (**a**). Baseline sensor representation. (**b**). Analysis of Renal Cancer Sample. We can see the baseline sensor representation, purge chamber with negative pressure and analysis of the sample. The colored lines represent the response of the 32 sensors.

We reported a brief graphical abstract of this study in Figure 3. A representative graphical image of the sensors’ response curves during the EN analysis was presented in Figure 4.

## 4. Discussion

Worldwide, health care systems have been heavily burdened by oncological diseases in terms of both lost lives and public costs. For this reason, more and more efforts have been made to develop strategies aimed at pursuing oncological mass screening and early detection of cancer. It is well known that patients who are diagnosed with an early-stage disease will have higher long-term overall survival and better disease-free survival rates, with a significant reduction in healthcare costs [19,20,21]. An early diagnose should also play a noticeable impact in the surgical setting. An early detection of kidney cancer would expand indications to nephron sparing surgery, minimizing ischemia time and the use of super-selective embolization, with the consequent benefit in terms of renal function [22,23,24]. Although technological advances have identified several blood and urine biomarkers as predictors of RCC diagnosis [25,26,27], none of them have been found reliable enough to be used in a clinical setting [28]. Therefore, the need for innovative diagnostic tests that are not only inexpensive and non-invasive, but at the same time sensitive and reliable, it still remains present. A careful estimation of potential new tools or molecules that can provide early diagnosis of RCC remains a translational research area that needs to be explored.

In RCC, the onco-metabolites, once aberrantly accumulated, contribute to tumorigenesis and can influence tumour phenotype and progression; for this reason, renal cancer is commonly considered a “cellular metabolism disease” [29]. Many studies, therefore, hypothesize that a metabolomics approach may prove itself useful in obtaining an early diagnosis of RCC. To investigate urinary volatile metabolites, we set out to collect urine samples which represent the gold standard for metabolomics studies in urinary tract diseases, in order to obtain a molecular platform from which to evaluate potential urinary biomarkers. This was possible because urine is a bio-fluid that is easy to collect, and also to store for successive analyses.

In this regard, the volatilome, that is, the volatile fraction of the metabolome, plays a crucial role in the scientific panorama thanks to the simplicity of the samples’ collection, the non-invasive nature of the measurements and the effective availability of analytical methods. Furthermore, there is already a range of evidence linking patterns of volatile organic compounds (VOCs) to a wide range of biological phenomena observable in vivo and in vitro [30,31]. It is currently possible to obtain an in-depth analysis of the volatilome composition through various instrumental techniques, among which the gas chromatography and mass spectrometers stand out. On the other hand, technological progress has produced new portable and easy-to-use tools based on arrays of sensors (so-called electronic noses). These devices, being able to analyse different human samples such as sweat, breath and urine, have been widely proven to be sensitive and selective tools in identifying diseases [32].

Metabolic alterations in the VOCs’ composition and concentration caused by cancer pathophysiology may generate a cancer VOC profile, especially through cell membrane peroxidation mechanisms [33]. The tumours produce altered VOCs compounds that are easily released into the atmosphere through the major body fluids such as saliva, sweat, breath and urine. Renal cancer cells are metabolically very active and produce large amounts of these compounds that can easily escape from tumour cells and localize in the urinary space, making urine an ideal substrate for investigating metabolomic biomarkers in RCC [34].

As far back as 400 BC, Hippocrates performed deep investigations into the diagnostic utility of body odours, reporting different specific odours released from urine in association with specific pathological conditions (infections, stones, diabetes) [35]. In consideration of the large number of constituents and its highly variable composition, urine is thought to be an extremely complex organic fluid from a chemical perspective. In fact, it is easy to verify that urine composition changes in relation to several factors such as age, gender, smoking, food habits, hormonal status, physical and sexual activity and the presence of distinct diseases [36].

Thanks to their exceptional olfactory acuity, dogs are able to recognize even small quantities of organic compounds with a characteristic odour [37]. This is the rationale behind their use in anti-drug, anti-terrorism and anti-counterfeiting operations or in the search for people lost in the mountains. However, the initial insight that dogs can recognize malignant tumours using their olfaction belongs to Williams and Pembroke in 1989 [38]. Since then, the olfactory power of trained sniffer dogs has been the centre of numerous studies aimed at detecting different types of cancer in humans (lung, ovarian, breast and melanoma), but unfortunately discrepant results have been obtained. In the specific context of urological cancers, sniffer-trained dogs have been successfully tested in the diagnosis of bladder and prostate cancer, but not yet in kidney cancers exhaustively. For example, the efficacy of trained dogs’ olfaction in detecting prostate cancer from human urine samples was first reported in 2010 by Cornu et al., with sensitivity and specificity rates around 91%. However, the small sample size (one dog and 66 patients) compromised the reliability of the study [39]. Similarly, in 2015, the study conducted by Taverna et al. reported in the same clinical setting (prostate cancer diagnosis) an accuracy of 97% with two trained dogs [40]. Lastly, Willis et al. tested six dogs in the bladder cancer context, reporting a successful diagnosis in 41% of the cancer cohort (95% CIs 23–58%) [41].

However, it must be underlined that renal function, diet or drug medications can be misleading factors in the olfactory ability of the trained dogs. This aspect represents a major drawback that unites all these studies. Unfortunately, all works involving dogs have obvious limitations: (1) dogs need adequate and lengthy training by a professional team, which is expensive and time-consuming; (2) the dog’s breed and the specific training methodology applied can affect the overall detection accuracy; and (3) dogs are not able to work reliably for many consecutive hours. Concerning the poor reliability and reproducibility on a large-scale of the canine model, the application of dog units in routine clinical practice is severely inhibited. Nevertheless, these findings underlined the importance of the potential role played by VOC profiling in the clinical diagnostic context.

For this reason, strong efforts have been undertaken by the scientific community worldwide to develop diagnostic tests capable of exploiting VOCs analysis. In the current range of diagnostic instruments aimed at identifying VOCs in a brief time, the electronic nose appears to be a cheap, portable and easy-to-use tool, that can be used without the aid of specialized technical personnel. The literature demonstrates that the e-Nose has already been successfully used in clinical practice to identify bacterial cultures or to diagnose kidney disease, diabetes or urinary tract infections [42]. Urine, being particularly rich in metabolites and easy to store, has quickly become the preferred source of VOCs, being easily collected in large quantities for clinical trials. Specific molecular VOC signatures are hypothesized to be associated with the presence of cancer, and for this reason the analysis of volatile organic compounds using artificial olfactory systems has emerged as a new non-invasive method for the early identification of tumours in biological samples [7]. Indeed, in recent years, more and more researchers have focused on the VOCs’ profiling. In this regard, we reported some interesting results: Altomare et al. [43] showed how the respiratory VOCs pattern in colorectal cancer patients was different from healthy controls; Ikeda et al. [44] suggested, for gastric cancer, an innovative diagnostic approach based on serum VOC profiling; Wu et al. [45] identified a unique urinary VOC profile for hepatocellular carcinoma patients; Filipiak et al. [46] proved that lung cancer-derived cells possessed specific volatile compounds. Taken together, these studies suggest that different cancers can provide different associated VOCs profiles. In the clinical setting of renal cancer by e-Nose, few of these studies have been conducted. In 2017, Nakhleh found that 33 patients with renal cancer were well recognized by breath analysis with an Artificially Intelligent Nanoarray similar to e-Nose, able to capture air VOCs, in comparison to healthy air samples [47]. In 2018, Wang compared the urinary VOC profiles of 22 RCC patients with 25 healthy subjects [48]. In 2021, Pinto et al. analysed the urinary VOCs profile of 75 renal cancer patients and 75 healthy controls, hypothesizing the existence of a volatile molecular signature characteristic for RCC [49].

As far as we have ascertained from the literature, this article is the first, extensive study on urinary VOCs profiling by electronic nose in the renal cancer setting. The present study showed, for CDA analysis, a CVA of 81.7% with a sensitivity and specificity of 71.8% and 89.4%, respectively. The discrimination accuracy of the model at the ROC analysis was 0.85. These results were confirmed by repeated analysis performed in a second set of independent urine samples (CVA 81.3%, AUC 0.85). The high positive (84.04%) and negative (80.37%) predictive values obtained in this first pilot study allowed the definition of the e-Nose as a promising screening tool in a renal cancer context, although the present findings need further clinical validations.

Since the e-Nose has demonstrated a high specificity, this aspect makes it a suitable tool to resolve some problematic questions related to the diagnostic procedures, such as patients’ overtreatment, possible complications associated with invasive procedures and high costs for the national health systems. Furthermore, the simple technology and the interesting results obtained configure the e-Nose as a promising tool in the early detection of renal cancer in the context of population screenings. Fundamental features for the clinical use of this innovative tool are the low cost, the non-invasive nature and the wide reproducibility in clinical settings, but we cannot fail to consider that, although the idea of introducing the e-Nose as a routine diagnostic test in clinical practice is fascinating, there are still different obstacles. We intend to discuss the three main limitations: (1) some exogenous factors such as medications, diet, smoking or alcohol consumption could contaminate the urine sample. To avoid the artifacts’ generation, a careful selection and separation of endogenous from exogenous VOCs is a mandatory step [50]. (2) e-Nose technology is unable to identify and quantify every single compound found in a sample; however, it can reveal specific molecular patterns. (3) Different bodily fluids can present varied concentrations and quantities of specific VOC patterns; this evidence implies the possible existence of several sets of volatile biomarkers in the different bodily fluids, all related to the same pathology. Therefore, the indispensable purpose of future scientific projects will be to recognize as many volatile compounds as possible, all referrable to the same molecular complex which, in turn, can be identified as a novel biomarker for RCC diagnosis or an innovative molecular target for systemic treatments. In relation to the present study, we recognize as possible limitations the monocentric design and the lack of an external validation cohort. A final consideration is that, today, radiological examinations such as TC or MRI scan show, surely, better results in terms of sensitivity and specificity compared to the results obtained in this study. However, while acknowledging CT and MRI as the gold standards for renal cancer detection, they are very expensive and time consuming exams [51].

### Clinical Translation

Despite the acknowledged limitations, the study’s results demonstrate that urinary VOC analysis by e-Nose can be considered an effective, non-invasive diagnostic tool to be used as a rapid mass screening test in renal carcinoma. Thanks to its characteristics of reproducibility, low cost and ease of use, this test could complement and enhance the normal diagnostic renal setting more economically than CT or MRI exams and less invasively than renal biopsies.

## 5. Conclusions

Although it was a single-centre study, we conducted the first extensive investigation into urinary volatilome profiling by an electronic nose in the field of renal carcinoma. Our results show the electronic nose as a valid tool capable of discriminating the urinary smell-prints of RCa patients from those of healthy controls, outperforming experiments conducted in past with trained sniffer dogs in a urological setting. In conclusion, we propose the urinary volatilome analysis by e-Nose as a promising clinical diagnostic tool in the field of RCC diagnosis, mainly due to its ease of use, non-invasive nature, low cost and wide reproducibility compared to initial studies based on trained animals.

## Figures and Tables

**Table 1:** Clinical features of the study population.

	RCa Cohort	HC Cohort	*p*–Value
N	110	142	
Age (years), mean ± SD (range)	64.53 ± 11.72 (36–86)	63.05 ± 14.27 (40–99)	0.378
Smokers, N (%)	29 (26.3%)	31 (21.8%)	0.404
Comorbidities, N (%)			
- Arterial hypertension	70 (63.6%)	75 (52.8%)	0.202
- History of AMI	5 (4.5%)	2 (1.4%)	0.133
- COPD	4 (2.6%)	3 (2.1%)	0.74
- Dyslipidemia	31 (28.1%)	29 (20.4%)	0.152

**Table 2:** Pathological Renal Tumour Classification.

TNM Stage	n. (%)	ISUP Grade	n. (%)
T1a	46 (30.6)	1	8
T1b	30 (20)	2	55
T2a	5 (3.3)	3	22
T2b	5 (3.3)	4	4
T3a	23 (15.3)	(only ccRCC)	89
T3b	1 (0.6)		
T3c	0 (0)		
T4	0 (0)		
Total	110 (73.3)		

**Table 3:** Group classification.

		Expected Group Membership		
	Group	HC	RCa	Total
Count	HC	127	15	142
	RCa	31	79	110
%	HC	89.4	10.6	100
	RCa	28.2	71.8	100

Based on 2D-PCA, 79/110 and 127/142 cases were correctly identified in the RCa and healthy control cohorts, respectively. For the RCa group, CDA analysis shows a correct classification in 81.7% of cases (*p* < 0.001), with SE of 71.8%, SP 89.4%, PPV 84.04% and NPV 80.37%.

## Data Availability

All data generated or analysed during this study are included in this published article. The support data can be download at URL https://drive.google.com/file/d/1mRxfhhaXb30eka2k0NseREIaevjt8KV2/view?usp=sharing (accessed on 10 February 2023).

## Abbreviations

EN or e-Nose	Electronic nose
VOCs	Volatile organic compounds
RCC	Renal cell carcinoma
PCA	Principal component analysis
CDA	Canonical discriminant analysis
CVA	Cross validated accuracy
ROC	Receiver operator curve
PPV	Positive prognostic value
NPV	Negative prognostic value
HC	Healthy control group
RCa	Renal cancer group
